# A prospective study of high dose sedation for rapid tranquilisation of acute behavioural disturbance in an acute mental health unit

**DOI:** 10.1186/1471-244X-13-225

**Published:** 2013-09-18

**Authors:** Leonie Calver, Vincent Drinkwater, Geoffrey K Isbister

**Affiliations:** 1 Discipline of Clinical Pharmacology, University of Newcastle, New South Wales, Australia; 2 Department of Clinical Toxicology and Pharmacology, Calvary Mater Edith St, Waratah, Newcastle, NSW, Australia; 3 Hunter New England Mental Health Centre, Psychiatric Emergency Services, Newcastle, Australia

**Keywords:** Violence, Sedation, Acute psychiatric unit, Droperidol, Benzodiazepine, Antipsychotic

## Abstract

**Background:**

Acute behavioural disturbance (ABD) is a common problem in psychiatry and both physical restraint and involuntary parenteral sedation are often required to control patients. Although guidelines are available, clinical practice is often guided by experience and there is little agreement on which drugs should be first-line treatment for rapid tranquilisation. This study aimed to investigate sedation for ABD in an acute mental healthcare unit, including the effectiveness and safety of high dose sedation.

**Methods:**

A prospective study of parenteral sedation for ABD in mental health patients was conducted from July 2010 to June 2011. Drug administration (type, dose, additional doses), time to sedation, vital signs and adverse effects were recorded. High dose parenteral sedation was defined as greater than the equivalent of 10 mg midazolam, droperidol or haloperidol (alone or in combination), compared to patients receiving 10 mg or less (normal dose). Effective sedation was defined as a fall in the sedation assessment tool score by two or a score of zero or less. Outcomes included frequency of adverse drug effects, time to sedation/tranquilisation and use of additional sedation.

**Results:**

Parenteral sedation was given in 171 cases. A single drug was given in 96 (56%), including droperidol (74), midazolam (19) and haloperidol (3). Effective sedation occurred in 157 patients (92%), and the median time to sedation was 20 min (Range: 5 to 100 min). The median time to sedation for 93 patients receiving high dose sedation was 20 min (5-90 min) compared to 20 min (5-100 min; p = 0.92) for 78 patients receiving normal dose sedation. Adverse effects occurred in 16 patients (9%); hypotension (14), oxygen desaturation (1), hypotension and oxygen desaturation (1). There were more adverse effects in the high dose sedation group compared to the normal dose group [11/93 (12%) vs. 5/78 (6%); p = 0.3]. Additional sedation was given in 9 of 171 patients (5%), seven in the high dose and two in the normal dose groups.

**Conclusions:**

Large initial doses of sedative drugs were used for ABD in just over half of cases and additional sedation was uncommon. High dose sedation did not result in more rapid or effective sedation but was associated with more adverse effects.

## Background

Acute behavioural disturbance (ABD) is a common occurrence in the healthcare setting of acute psychiatry [1,2]. Both physical restraint and involuntary parenteral sedation are often required to control patients with ABD. The overall goal is to achieve rapid sedation or tranquilisation to prevent injury to the patient, other patients or staff, whilst minimizing adverse drug effects. When all other strategies such as verbal de-escalation have failed to manage the ABD, parenteral sedation is recommended to prevent distress and reduce harm [3-5].

Although many prescriptive guidelines are available, clinical practice is often primarily guided by experience as there is little agreement on which drugs should be used as first-line treatment for rapid tranquilisation. There is general consensus in the literature that benzodiazepines alone, an antipsychotic alone or a combination of the two are the first line agents for sedation in patients with ABD [1]. Clinical practice guidelines are reasonably consistent in recommending doses of 5 mg to 10 mg of a typical antipsychotic and 5 to 10 mg of midazolam or diazepam (or 2 mg of lorazepam) when used in combination [1,6-9]. However, larger doses are often used, exceeding doses recommended in many clinical practice guidelines [1,9] and the recommendations of the British National Formulary [10]. This is acknowledged by expert clinical opinion [1,11,12].

This study aimed to investigate the types, doses and frequency of drugs used for sedation of ABD in an acute mental health unit, and the frequency of adverse drug effects. We hypothesized that larger doses of sedation might increase the frequency of adverse effects and may reduce the requirement for additional sedation.

## Methods

This was a prospective observational study of patients with ABD in an acute mental healthcare unit who required parenteral sedation and physical restraint to protect themselves and/or others. We measured the time to sedation, frequency of adverse effects and use of additional sedation.

The study was undertaken from July 2010 to June 2011 in an eight bed acute mental healthcare unit in a tertiary specialist mental health facility with a 90% occupancy rate. Ethics approval was obtained from the local Human Research Ethics Committee. Consent was waived because of the requirement for immediate treatment and patients’ lack of decision-making capacity to consent to medical treatment being given as a duty of care.

The study included all patients administered parenteral sedation for ABD in the acute mental healthcare unit. Admissions to the unit are from the psychiatric emergency care center and are referred from general practitioners, regional hospitals or other units within the institution.

All patients with ABD in the acute mental healthcare unit who did not calm with verbal de-escalation or oral medication and who required physical restraint and parenteral sedation were included. The choice of drug or drugs and the doses administered were determined by the treating clinician. Parenteral sedation was given by initially physically restraining the patient to administer the medications. The patient was then put in a seclusion room and was not mechanically restrained. All patients were involuntary admissions.

Vital signs were recorded every 10 minutes for the first hour then half hourly until the patient settled. A number of the observations were recorded remotely, including the respiratory rate and the level of agitation. Remote observations were commenced from the onset of the ABD until it was considered safe to approach the patient and record vital signs including heart rate, blood pressure, oxygen saturation and respiratory rate. The level of sedation and agitation was recorded using the sedation assessment tool (SAT; Table 1) [13]. The SAT scores the patient from +3 (physically violent) to -3 (unconscious) and allows rapid assessment before and after sedative medication is given. A initial score of +2 or +3 was required and almost always reported in patients requiring physical restraint and parental sedation. We have previously defined effective rapid sedation or tranquilisation as a fall in the score by two levels or a score of zero or less [13-15]. An additional dose of sedative medication was encouraged by the senior medical staff after 30 minutes if there was no response to the first drug given. If the patient did not sedate after 120 minutes they were considered to have failed sedation. The patient was observed for extrapyramidal side-effects and any additional medications were recorded.

**Table 1:** Sedation Assessment Tool: SAT

**SCORE**	**RESPONSIVENESS**	**SPEECH**
**+3**	Combative, violent, out of control	Continual loud outbursts
**+2**	Very anxious and agitated	Loud outbursts
**+1**	Anxious and restless	Normal
**0**	Responds easily to name, speaks in normal tone	Normal
**-1**	Responds only if name is called loudly	Slurring or prominent slowing
**-2**	Physical stimulation	Few recognisable words
**-3**	No response to stimulation	Nil

At the commencement of the study a previously developed ABD chart was introduced into the acute mental healthcare unit. The ABD chart is part of the medical record and is used to record the level of agitation and sedation with the SAT, vital signs and any adverse effects that occur. The use of the form allowed the simultaneous use of the information for research which was obtained for clinical care of the patient. The following data were then extracted from the ABD chart and medical record: age, sex, medication used including time of administration, dose and additional sedation given. For this study high dose parenteral sedation was defined as a dose greater than the equivalent of 10 mg of midazolam, 10 mg of droperidol or 10 mg of haloperidol, whether as a single agent or a combination of these three drugs. This was based on a controlled trial that compared droperidol (10 mg) versus midazolam (10 mg), versus the combination of droperidol (5 mg) and midazolam (5 mg) [14], and the fact that these were the commonest drugs used in the institution during the study. Patients receiving equal to or less than 10 mg of these three drugs were classified as the normal dose group.

The outcomes for this study were the time to sedation/tranquilisation defined as a fall in the SAT score by two levels or a score of zero or less; the proportion of patients with adverse drug effects defined as a respiratory rate less than 12 breaths per min, systolic blood pressure less than 90 mmHg, oxygen saturation less than 90% or the presence of extrapyramidal side-effects; and the use of additional sedation. The outcomes were compared between patients receiving high dose parenteral sedation and those receiving normal or a lower dose.

Medians and interquartile ranges (IQR) are reported for all continuous variables. Percentages are reported for dichotomous outcomes with 95% confidence intervals (CI). Dichotomous outcomes were compared using Fisher’s exact test. All analyses and graphics were done in GraphPad Prism version 5.03 for Windows, GraphPad Software, San Diego California USA, http://www.graphpad.com.

## Results

There were 171 occasions of patients with ABD requiring parenteral sedation in 95 patients during the 12 month period. The median age of the patients was 40 years (15 to 80 yr) and 121 patients were male. The median SAT score prior to sedation was 2 (IQR: 2 to 3). A single drug was administered on 97 occasions and was most commonly droperidol (74; median dose 10 mg; range 5 to 30 mg), then midazolam (19; median dose 10 mg; range 5 to 15 mg) and haloperidol (3; all 10 mg). Combinations of drugs were used in the remaining 75 occasions with the combination of droperidol (10 mg) and midazolam (10 mg) being the most common on 61 occasions. Table 2 lists the different combinations of drugs, the range of drug doses and the frequency used. Ninety three patients (54%) were given more than the equivalent of 10 mg droperidol/midazolam (high dose parenteral sedation), and the majority of these were patients were given 10 mg of either droperidol or haloperidol in combination with 10 mg of midazolam (Table 2). There was no significant difference in age, sex and initial SAT score between patients receiving high dose sedation versus normal sedation dose.

**Table 2:** Details of the initial drug type and dose for all 171 episodes and the adverse effects and median time to sedation for each drug type/combination

**Drug type**	**Initial drug**	**Dose range (mg)**	**Adverse effects**	**Median time to sedation (min)**
			**N = 16**	
Droperidol	74	5 to 30	Hypotension (3)	20
Midazolam	19	5 to 15	Hypotension (1)	20
Haloperidol	3	10	0	30
Midazolam + droperidol	61	5 to 15 +	Hypotension (8)	25
		5 to 25	Desaturation/hypotension (1)	
Midazolam + haloperidol	12	5 to 10 + 10	Hypotension (2)	10
			Desaturation (1)	
Droperidol/lorazepam	1	2.5 + 2	0	15
Lorazepam	1	2		40

**Figure 1:**
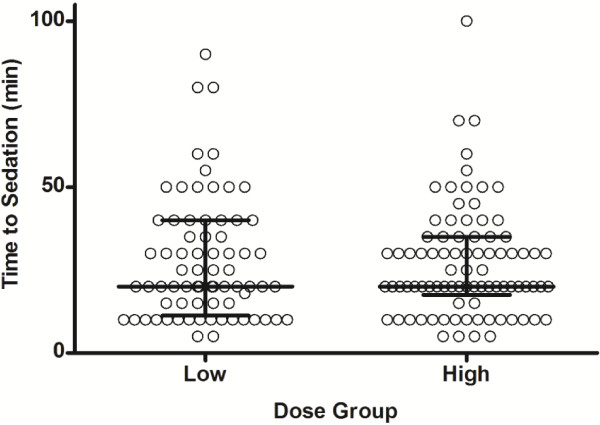
The time to sedation for the high dose group compared to the low dose group.

Effective sedation was achieved in 157 patients, and the median time to sedation was 20 min (IQR: 15 to 35 min; Range: 5 to 100 min). The median time to sedation for high dose sedation was 20 minutes (IQR: 18 to 35 min; Range: 5 to 100 min), and 20 minutes (IQR: 11 to 40 min; Range: 5 to 90 min) for normal dose sedation (Figure 1). The remaining 14 patients were not sedated with the initial dose of sedation; eight given high dose and six given normal dose.

**Figure 2:**
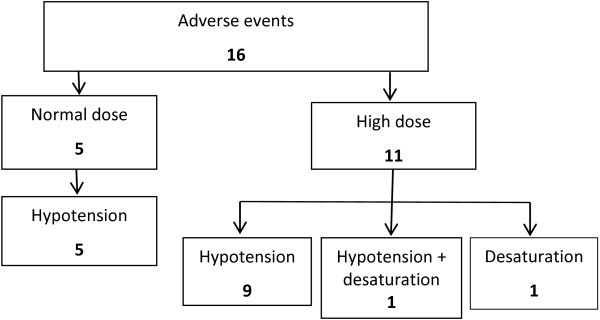
Adverse effects based on high or normal dose sedation and the types of adverse effects.

Adverse effects occurred in 16 patients (9%), including hypotension (14), oxygen de-saturation (1), hypotension and oxygen de-saturation (1) (Table 2). The frequency of adverse effects was higher for the high dose group compared to the normal dose group [11/93 (12%) vs. 5/78 (6%); p = 0.3] (Figure 2). Of the 14 patients not sedated none received additional sedation. Additional sedation was administered to 9 of the 171 patients (5%), seven in the high dose group and two in the normal dose group.

## Discussion

The study found that large initial doses of parenteral sedation are commonly used in the treatment of ABD in the acute mental health setting. The higher dose parenteral sedation was not associated with a shorter time to sedation, but was associated with a higher frequency of adverse effects. Of the 16 adverse drug effects that occurred, almost two-thirds occurred in the patients who received high dose parenteral sedation. The study also showed that the additional sedation was rarely used in both groups.

The high dose parenteral sedation of greater than 10 mg of droperidol, haloperidol or midazolam alone or in combination, is more than the recommended dose in clinical practice guidelines [1,9]. It is also greater than the doses from randomized controlled trials in the acute psychiatric setting where initial doses ranged from 5 to 10 mg of droperidol or midazolam/haloperidol or equivalent amounts of other sedative drugs [16-19].

Unfortunately there is a lack of good evidence to support the effectiveness and safety of drugs used for rapid tranquilisaton of ABD in the acute psychiatry setting. This may be in part due to the difficulties in obtaining ethics approval to study this vulnerable patient population and the need to obtain consent from patients without the capacity to consent. Many trials only include patients with written consent [20,21] and require co-operation from the patients prior to recruitment in the form of performing tests and obtaining blood samples [21] which is rarely possible due to safety issues in patients with ABD. This means that trials in the mental health setting rarely include the most agitated patients and the treatment of this difficult group of patients with ABD is often guided by clinical experience rather than evidence from clinical trials. This may explain the disparity between what is in clinical guidelines based on the literature and what actually happens with the sedation of ABD in the clinical setting.

A difference found in this study compared to other studies of management of ABD in mental health [16-22] was the large initial doses of medication administered to patients, which was often a combination of medications. Combinations of medications, most commonly a benzodiazepine and an antipsychotic, reflects a common strategy in the acute psychiatry setting [6,23]. Although the literature supports the strategy of combining agents, it recommends that lower doses of each medication are used to reduce the risk of adverse effects [6,23]. However, in this study the use of combinations of drugs resulted in a larger total dose being administered in most patients. Importantly, the larger dose did not result in more rapid sedation, but did result in an increased frequency of adverse effects (Figure 2).

The frequency of adverse effects in this study may be an underestimate of the true frequency because of the difficulties in obtaining a complete set of vital signs in these dangerous patients. The inability to have immediate access to the patient due to the level of agitation makes the recording of vital signs and the detection of adverse effects more difficult in the mental health care setting. Only the respiratory rate and SAT could be recorded from outside the seclusion room until the patient was sedated sufficiently to allow the recording of blood pressure and oxygen saturations.

Additional sedation was rarely used in this study which is most likely due to the danger associated with approaching a violent patient on a second occasion once they are in seclusion. In this study 14 patients were not sedated after the initial dose, but additional sedation was only administered in nine patients. This differs to studies of sedation of ABD in the emergency department where 26% to 45% of patients are given additional sedation [14,24].

There were a number of limitations to the study including the non-randomised nature of the sample. This may have introduced bias because patients with more severe ABD may have been more likely to be given high dose sedation. However, there was no difference in the initial SAT score between patients in the high and normal dose sedation groups. The overall frequency of adverse effects in the study was low so the difference between the high and normal dose groups did not reach statistical significance. A larger study is required to confirm this finding.

## Conclusion

The study has shown that large initial doses of sedative drugs were used in just over half of cases of ABD in the mental health setting. High dose sedation did not result in more rapid or effective sedation than normal or lower doses of sedation. However, high dose sedation was associated with more adverse effects. Additional sedation was uncommon in all patients. This suggests there is no benefit and potential risk if large initial doses of sedation are given to patients with ABD. Doses recommended by the majority of guidelines should be used and larger doses of single agents or combinations of drugs should be avoided.

## Abbreviations

ABD: Acute behavioural disturbance; SAT: Sedation assessment tool; IQR: Interquartile range; CI: Confidence interval.

## Competing interests

The authors declare that they have no competing interests.

## Authors’ contributions

LC conceived the study, coordinated data collection and drafted the manuscript; VD participated in the design of the study and recruited patients. GI helped conceive the study, designed the study, performed the statistical analysis and helped to draft the manuscript. All authors read and approved the final manuscript.

## Pre-publication history

The pre-publication history for this paper can be accessed here:

http://www.biomedcentral.com/1471-244X/13/225/prepub

## References

[B1] National Institute of Clinical Excellence Violence: The short term management of distiurbed/violent Behaviourin the Psychiatric Patient Settings and the Emergency Departments 2005 London: Clinical Guideline

[B2] Cornaggia CM Beghi M Pavone F Barale F Aggression in psychiatry wards: a systematic review Psychiatry Res 2011 189 1 10 20 10.1016/j.psychres.2010.12.024 21236497

[B3] Stubbs B Physical injuries from restrictive interventions Psychiatr Serv 2009 60 3 406 10.1176/appi.ps.60.3.406 19252060

[B4] Allen MH Currier GW Hughes DH Reyes Harde M Docherty JP The Expert Consensus Guideline Series. Treatment of behavioral emergencies Postgrad Med 2001 Spec No 1 88 quiz 89-90 11500996

[B5] Brown S Chhina N Dye S Use of psychotropic medication in seven English psychiatric intensive care units The Psychiatrist 2010 34 130 135 10.1192/pb.bp.108.023762

[B6] Allen MH Currier GW Carpenter D Ross R Docherty JP The Expert Consensus Guideline Series. Treatment of behavioral emergencies 2005 J Psychiatr Pract 2005 11 5 112 16319571 10.1097/00131746-200511001-00002

[B7] Currier GW Trenton A Pharmacological treatment of psychotic agitation CNS Drugs 2002 16 4 219 228 10.2165/00023210-200216040-00002 11945106

[B8] Brattaglia J Moss S Rush J Kang J Mendoza R Leedom L Dubin W McGlynn C Goodman L Haliperidol, Lorazepam, or both for Psychotic Agitation? A Multiceter,Prospective, Double-Blind, Emergency Department Study Am J Emerg Med 1997 15 4 10.1016/s0735-6757(97)90119-4 9217519

[B9] Mental Health Drug and Alcohol Office Mental Health for Emergency Departments-A Reference Guide 2009 Sydney: NSW Department of Health

[B10] British Medical Association and the Royal Pharmaceutical Society of Great Britain British National Formulary 2001 London

[B11] Mantovani C Migon MN Alheira FV Del-Ben CM Management of the violent or agitated patient Rev Bras Psiquiatr 2010 32 Suppl 2 S96 103 21140077 10.1590/s1516-44462010000600006

[B12] Chakrabarti A Whicher E Morrison M Douglas-Hall P 'As required' medication regimens for seriously mentally ill people in hospital Cochrane Database Syst Rev 2007 3 CD003441 10.1002/14651858.CD003441.pub2 17636723

[B13] Calver LA Stokes B Isbister GK Sedation assessment tool to score acute behavioural disturbance in the emergency department Emerg Med Australas 2011 23 6 732 740 10.1111/j.1742-6723.2011.01484.x 22151672

[B14] Isbister GK Calver LA Page CB Stokes B Bryant JL Downes MA Randomized controlled trial of intramuscular droperidol versus midazolam for violence and acute behavioral disturbance: the DORM study Ann Emerg Med 2010 56 4) 392 401 e391 20868907 10.1016/j.annemergmed.2010.05.037

[B15] Calver L Downes M Page C Bryant J Isbister G The impact of a standardised intramuscular sedation protocol for acute behavioural disturbance in the emergency department BMC Emerg Med 2010 10 1 14 10.1186/1471-227X-10-14 20584282 PMC2906413

[B16] Meehan K Zhang F David S Tohen M Janicak P Small J Koch M Rizk R Walker D Tran P A double-blind, randomized comparison of the efficacy and safety of intramuscular injections of olanzapine, lorazepam, or placebo in treating acutely agitated patients diagnosed with bipolar mania J Clin Psychopharmacol 2001 21 4 389 397 10.1097/00004714-200108000-00006 11476123

[B17] Breier A Meehan K Birkett M David S Ferchland I Sutton V Taylor CC Palmer R Dossenbach M Kiesler G A double-blind, placebo-controlled dose-response comparison of intramuscular olanzapine and haloperidol in the treatment of acute agitation in schizophrenia Arch Gen Psychiatry 2002 59 5 441 448 10.1001/archpsyc.59.5.441 11982448

[B18] Meehan KM Wang H David SR Nisivoccia JR Jones B Beasley CM Jr Feldman PD Mintzer JE Beckett LM Breier A Comparison of rapidly acting intramuscular olanzapine, lorazepam, and placebo: a double-blind, randomized study in acutely agitated patients with dementia Neuropsychopharmacology 2002 26 4 494 504 10.1016/S0893-133X(01)00365-7 11927174

[B19] Resnick M Burton BT Droperidol vs. haloperidol in the initial management of acutely agitated patients J Clin Psychiatry 1984 45 7 298 299 6376480

[B20] Wright P Lindborg SR Birkett M Meehan K Jones B Alaka K Ferchland-Howe I Pickard A Taylor CC Roth J Intramuscular olanzapine and intramuscular haloperidol in acute schizophrenia: antipsychotic efficacy and extrapyramidal safety during the first 24 hours of treatment Can J Psychiatry 2003 48 11 716 721 14733451 10.1177/070674370304801102

[B21] Lesem MD Tran Johnson TK Riesenberg RA Feifel D Allen MH Fishman R Spyker DA Kehne JH Cassella V Rapid acute treatment of agitation in individuals with schizophrenia: multicentre, randomised, placebo-controlled study of inhaled loxapine Br J Psychiatry 2011 198 51 58 10.1192/bjp.bp.110.081513 21200077

[B22] Wright P Birkett M David SR Meehan K Ferchland I Alaka KJ Saunders JC Krueger J Bradley P San L Double-blind, placebo-controlled comparison of intramuscular olanzapine and intramuscular haloperidol in the treatment of acute agitation in schizophrenia Am J Psychiatry 2001 158 7 1149 1151 10.1176/appi.ajp.158.7.1149 11431240

[B23] Binder RL EMD Contemporary practices in managing acute violent patients in 20 psychiatric emergency rooms Psychiatr Serv 1999 50 12 1553 1554 10577870 10.1176/ps.50.12.1553

[B24] Martel ML Ziprasidone for sedation of the agitated ED patient Am J Emerg Med 2004 22 3 238 15138973 10.1016/j.ajem.2004.02.027

